# Six months SARS-CoV-2 serology in a cohort of mRNA vaccinated subjects over 90 years old

**DOI:** 10.1038/s41598-022-15148-z

**Published:** 2022-07-20

**Authors:** Rossella Tomaiuolo, Chiara Di Resta, Marco Viganò, Giuseppe Banfi, Cristina Russo, Giulia Linardos, Stefania Ranno, Carlo Federico Perno, Francesco Giuffrida

**Affiliations:** 1 Vita-Salute San Raffaele University, Milan, Italy; 2 IRCCS Galeazzi Orthopaedic Institute, Milan, Italy; 3 IRCCS Bambino Gesù Children’s Hospital, Rome, Italy; 4 Cooperativa OSA, Rome, Italy

**Keywords:** Geriatrics, Epidemiology

## Abstract

Ageing is associated with a progressive decline and remodelling of the immune system. Also, the efficacy of COVID-19 vaccines has been observed to depend on subjects’ age. The post-vaccination data about patients aged > 90 years old is scarcely represented in the literature. The antibody titre profiles of elderly vaccinated subjects (age > 90 years old) were evaluated and compared with profiles obtained in a younger population (age 23–69 years old). To the best of our knowledge, this is the first report providing post-vaccination serological data in subjects aged 90 + years old. This study suggests that distinct SARS-CoV-2 viral-specific antibody response profiles vary based on anti-N serostatus, age, and sex in the very elderly adults. The data obtained could impact the organisation of the vaccination campaign (i.e., prioritisation strategies, administration of additional doses) and the factors that facilitate intentions to receive the vaccination among elderly adults (i.e., vaccine effectiveness).

## Introduction

Coronavirus disease 2019 (COVID-19), caused by severe acute respiratory syndrome coronavirus 2 (SARS-CoV-2), was described in December 2019, with more than 412 million confirmed cases, including more than 5 million deaths, as of 15 February 2022^1^. The mortality rate appears to be higher for elderly patients: 18.5% for patients between 70 and 79 years old and about 25% for patients older than 80 years. Conversely, it was < 1% below 50 years old^1^. Elderly adults are at higher risk of developing severe symptoms and morbidity of COVID-19^2^, as ageing is associated with a progressive decline and remodelling of the immune system, resulting in an increased risk of severe outcomes from infectious diseases^3,4^. Indeed, people aged 65 years old or more account for most influenza-related hospitalisations and over 70% of all influenza-related deaths^5^. Furthermore, among individuals whose age is 80 or more, males are more likely than females to be hospitalised and die due to influenza virus infections^6,7^.

The efficacy of COVID-19 vaccines has been observed to depend on subjects’ age, both in terms of protection against infection, hospitalisation and death, as well as in terms of serum antibody titre against SARS-CoV-2 spike protein^8–11^; on the other hand, more frequent adverse reactions were associated with younger age^12,13^. Furthermore, the immune response is influenced by sex and gender as sex hormones differentially modulate immune responses^12,13^. While these differences have been described for several age categories, the post-vaccination data about patients aged > 90 years old is scarcely represented in the literature.

To this aim, this study evaluates the antibody titres after the vaccination against SARS-CoV-2 in very elderly subjects (age > 90 years old) and compares it to data obtained in a younger population (age 23–69 years old) in the context of a study conducted in Italy. The data obtained could impact the organisation of the vaccination campaign (i.e., prioritisation strategies, administration of additional doses) and the factors that facilitate intentions to receive the vaccination among elderly adults (i.e., vaccine effectiveness).

## Materials and methods

### Studied cohort and data sources

This cross-sectional study used SARS-CoV-2 antibody test results from general (n. 1114 of 23–69 years old) and a very elderly population (n. 97 of 90–99 years old, named "+ 90 years old population" in the manuscript) after the vaccination. The data gathered in the COVIDIAGNOSTIX project were collected from December 2020 to September 2021. According to the approved protocol (CE:199/INT/2020) of the Ethical Review Board of IRCCS San Raffaele Hospital, written informed consent was obtained from all the participants.

The general population was composed of healthcare workers at IRCCS San Raffaele Hospital enrolled during the Italian COVID-19 vaccination campaign. The + 90 years old population includes subjects receiving home care assistance by the OSA Cooperative (Operatori Sanitari Associati ONLUS) for rehabilitation or medication purposes. The blood samples of the elderly adult population were collected by the staff of the OSA, participating as a partner in the COVIDIAGNOSTIX study. At the time of blood harvesting, the + 90 y/o cohort also underwent a rapid serological test. The blood samples were analysed at the Microbiology Laboratory of the Bambino Gesù Children’s Hospital, involved in the study. Sampling was performed in both populations 6 months after the administration of the two doses required for the completion of the vaccination cycle. The vaccine administered was the BNT162b2 mRNA COVID-19 Vaccine; thus, the response depends exclusively on the recipient.

### Methods

The antibody titre was tested in both study groups by the Elecsys Anti-SARS-CoV-2 assay (Roche, Basel, Switzerland) specific for the viral SARS-CoV-2 nucleocapsid (N) protein and by the Elecsys SARS-CoV-2-S (Roche, Basel, Switzerland) against the receptor-binding domain (RBD) of the viral spike protein (S-protein).

The Roche Elecsys Anti-SARS-CoV-2 run on the COBAS 601 platform (Roche, Basel, Switzerland); it is an electrochemiluminescence immunoassay (ECLIA) targeted on total immunoglobulins (IgTot) against the N-protein. As reported in the manufacturer’s datasheet (ref: 09289267501V0.6), the result is given as a cut-off index (COI) as well as in the form of qualitative results: for COI < 1.0, the sample is nonreactive and negative; for COI > 1.0, the sample is reactive and positive. The manufacturer indicated specificity (95% CI) of 99.80% and sensitivity (95% CI) of 99.5 14 days post-PCR confirmation^14^.

The Roche anti-SARS-CoV-2-S run on COBAS 601 platform (Roche, Basel, Switzerland); it is an electrochemiluminescence immunoassay (ECLIA) detecting total Immunoglobulins (IgTot: IgA, IgG and IgM) against the receptor-binding domain (RBD) of the viral S-protein. The quantification range is between 0.4 and 250.0 U/mL which is further extended to 2500.0 U/mL by a 1:10 dilution of the sample automatically performed by the instrument. Specificity and sensitivity (≥ 14 days after diagnosis) are 99.98% and 98.8%, respectively, when the manufacturer’s suggested cut-off of 0.8 U/mL is used^14^.

Individuals in the + 90 y/o group were also tested with the Diasorin test and SARS-CoV-2 Rapid Antibody test device (Roche Diagnostics, ID 09216448190). The Diasorin LIAISON SARS-CoV-2 Trimerics IgG (Diasorin, Saluggia, Italy) is a CLIA detecting IgG specific to the Trimeric Spike Glycoprotein. The quantification range is between 1.85 and 800 U/mL. Specificity and sensitivity (> 15 days after diagnosis) are 99.5% and 98.7%, respectively, when the manufacturer’s suggested cut-off ≥ 13 U/mL is used. The POCT SARS-CoV-2 Rapid Antibody test device (Roche Diagnostics, ID 09216448190) is a chromatographic immunoassay that detects antibodies (IgM/IgG) to SARS-CoV-2 in just 10 min. The SARS-CoV-2 IgM and IgG antibodies present in the sample interact with gold particle-labelled SARS-CoV-2 N and S antigens to form an antibody-antigen-gold-particle complex. This test was performed to give an immediate result to + 90 years old population. However, a positive test refers to the qualitative detection of antibodies but cannot provide any information on the level of antibody concentration in the sample.

A comparison of techniques was performed to demonstrate equivalence of the Elecsys SARS-CoV-2-S (Roche, Basel, Switzerland) and LIAISON SARS-CoV-2 Trimerics IgG (Diasorin, Saluggia, Italy) assays in reporting positive and negative results.

### Statistical analysis

Analysis was performed using R software v4.1.1 (R Core Team, Wien, Austria)^15^. Categorical data are expressed as absolute frequency and percentage. Continuous variables were tested for normality using the Shapiro–Wilk test and data are expressed as median and range/interquartile range or mean ± SD according to the results of this test, in case of non-normal and normal distribution, respectively. For the same reason, correlation analyses were performed using Spearman’s method. Receiver Operator Characteristics (ROC) curves were used to identify the anti-S antibody titre threshold maximising the Area Under Curve (AUC) (with Youden’s method) for the positive outcome of rapid IgG tests. The beta transformation was obtained by dividing the antibody titre for the maximum value (2500 U/mL). Betareg package for R was used to fit beta regression models ^16^. *P* values < 0.05 were considered statistically significant.

### Ethical statement

The study was conducted according to the guidelines of the Declaration of Helsinki, and approved by the Ethical Review Board of IRCCS San Raffaele Hospital (Protocol No. CE:199/INT/2020; date of approval December 23rd, 2020, approved by the IRCCS San Raffaele Hospital Ethical Review Board). Informed consent was obtained from all subjects involved in the study.

## Results

The two analysed cohorts of subjects aged 23–69 years old and + 90 years old include 1114 and 97 individuals. Proportions of females and anti-N seropositive subjects and follow-up time were similar between the two cohorts (Table 1). In both cases, females were more represented, while the prevalence of anti-N positivity was around 8–9% of subjects.

**Table 1:** Summary of subjects’ data.

	23–69 years old		+ 90 years old	
Total number of subjects	1114	97		
Gender	Females	Males	Females	Males
n (%)	732 (65.7%)	382 (34.3%)	66 (68.0%)	31 (32.0%)
Presence of anti-N antibodies	Yes	No	Yes	No
n (%)	91 (8.2%)	1023 (91.8%)	9 (9.3%)	88 (90.7%)
Months after administration of 2nd dose (mean ± SD)	6.2 ± 0.8		5.8 ± 1.1	

### Anti-spike antibody titre at 6 months after 2nd dose

Significant differences were observed in anti-S antibody titre (by the Roche Elecsys SARS-CoV-2-S) between anti-N seronegative and seropositive subjects in both cohorts, with medians of 587 (343–960) U/mL and 2500 (1375–2500) U/mL in the 23–69 y/o cohort (*p* < 0.001), respectively, while in the + 90 y/o cohort these values were 65 (27–188) U/mL) and 2500 (1534–2500) U/mL (*p* < 0.001).

**Figure 1:**
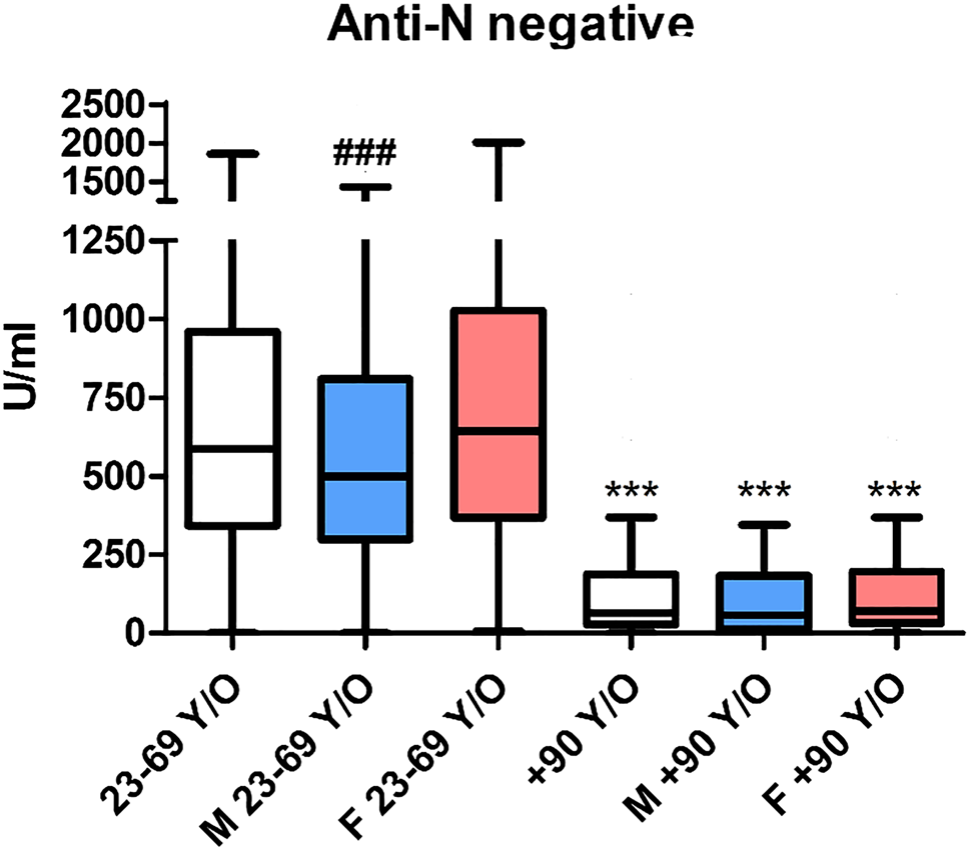
Anti-S antibody titre (by the Roche Elecsys SARS-CoV-2-S) in individuals seronegative for anti-N. The blue box (M) indicates male subjects, and the red one (F) indicates female subjects. ***p < 0.001 versus the correspondent color in the 23–69 y/o cohort; ### *p* < 0.001 versus females in the same cohort.

Considering seronegative subjects only, a significant reduction was observed between the two cohorts with an evident lower response in + 90 y/o subjects (*p* < 0.001) (Fig. 1). Differences between males and females were observed in the 23–69 y/o cohort only (*p* < 0.001), while subjects + 90 y/o, males and females, showed similar values. The age-related differences were observed also considering males and females (*p* < 0.001) separately.

**Figure 2:**
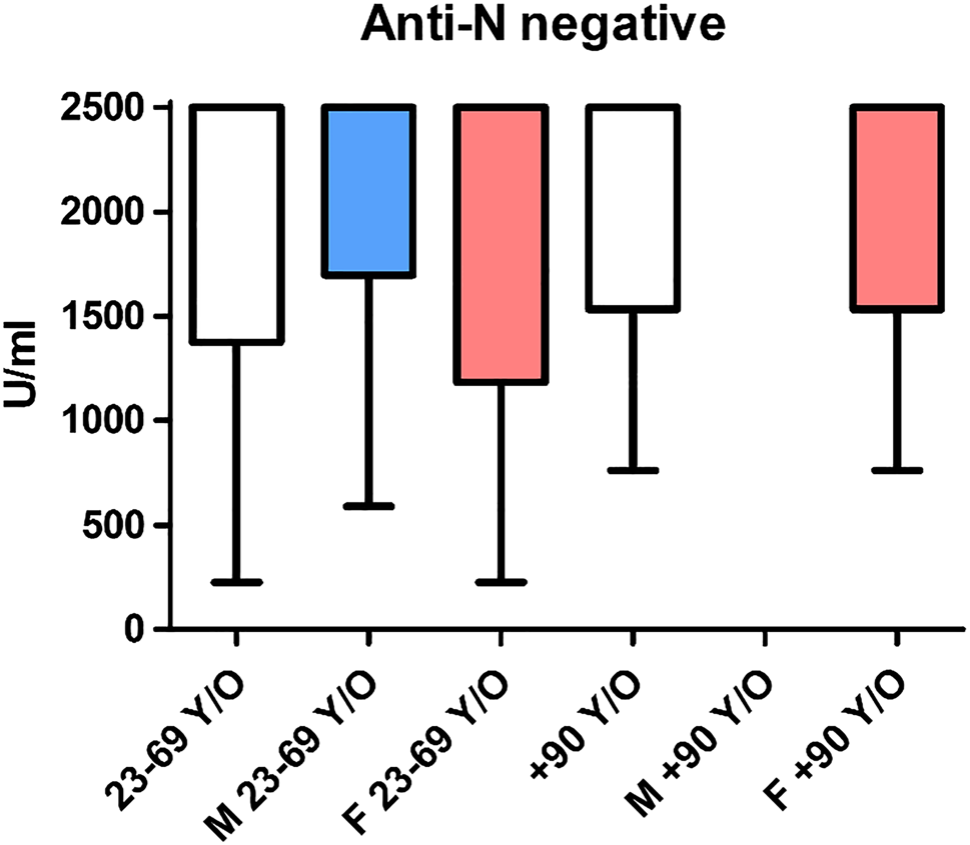
The anti-S antibody titre (by the Roche Elecsys SARS-CoV-2-S) in individuals seropositive for anti-N. The blue box (M) indicates male subjects, and the red one (F) indicates female subjects. No seropositive male subjects were observed in the + 90 y/o cohort.

Seropositive subjects were females in 60.4% of cases (n = 56) in the younger cohort, while no males were observed among the seropositive subjects in the + 90 y/o group, where only seven female subjects were identified. Thus, comparisons were possible in the female subset only, and no differences were identified between younger and older age groups (*p* = 0.526) (Fig. 2).

### Variables influencing the antibody titre 6 months after the 2nd dose

Using a beta distribution after appropriate linear transformation, it was possible to describe the anti-S antibody titre distribution at 6 months (Supplementary Figure S1). Beta regression models designed using antibody titre as the dependent response variable and age, anti-N positivity and gender as independent covariates allowed identifying a significant influence of all three variables in the determination of anti-S antibody titre (Table 2). In particular, according to Wald test statistic, the most critical factor in determining anti-S antibody titre was anti-N seropositivity, followed by age. The contribution of gender was significant but limited compared to the two other variables. Table 2 shows that the full model (anti-N serostatus + Age + Gender) possesses better properties than Reduced model 2 (without anti-N serostatus covariate). In contrast, the addition of gender produces a minimal effect on model performances.

**Table 2:** Summary of beta regression models.

	Full model	Reduced model 1	Reduced model 2
Formula	anti-S (U/mL) ~ anti-N serostatus + Age + Gender	anti-S (U/mL) ~ anti-N serostatus + Age	anti-S (U/mL) ~ Age + Gender
Intercept Estimate	− 8.15	− 8.17	− 7.90
Intercept Std. Error	0.06	0.06	0.07
Intercept Wald test statistics	127.00	− 129.09	− 116.17
Intercept *p* value	< 0.001	< 0.001	< 0.001
Anti-N serostatus + Estimate	0.98	0.97	
Anti-N serostatus + Std. Error	0.05	0.05	
Anti-N serostatus + Wald test statistics	19.47	19.28	
Anti-N serostatus + *p* value	< 0.001	< 0.001	
Age Estimate	− 0.02	− 0.02	− 0.02
Age Std. Error	0.001	0.001	0.001
Age Wald test statistics	− 15.06	− 15.09	− 15.74
Age p value	< 0.001	< 0.001	< 0.001
Gender M Estimate	− 0.09		− 0.04
Gender M Error	0.04		0.04
Gender M Wald test statistics	− 2.18		− 0.89
Gender M p-value	0.029		0.373
Log-likelihood	8726 on 5 Df	8723 on 4 Df	8617 on 4 Df
AIC	− 17,441.72	− 17,438.80	− 17,225.38
Pseudo R-squared	0.340	0.337	0.255

### Different tests for the evaluation of anti-spike antibody titre

Subjects in the + 90 y/o group were also tested with the Diasorin test and rapid antigenic serological tests. Similar differences between anti-N seronegative and seropositive subjects were observed with this test compared with the Roche test. A median of 42.5 (1.8–2080.0) UA/mL in anti-N seronegative samples and 2080.0 (355.0–2080.0) U/mL in seropositive subjects were observed.

In addition, these results correlate strongly with those from the Roche test, with a Spearman’s r equal to 0.833 (*p* < 0.001).

The ability of the rapid tests to identify IgG anti-S antibody was good. These tests resulted positive for serum antibody titre > 196.5 UA/mL (Roche) with sensitivity 100.0% and specificity 82.9% (AUC: 98.1%, CI95%: 87.8–100.0%). In addition, a correlation was observed between good test results and levels of positivity on rapid tests (slightly positive, positive, strongly positive), with medians equal to 809, 899 and 3263 UA/mL, respectively, for the three increasing levels.

## Discussion

The main finding of this study is that very elderly adults produce measurable amounts of anti-S antibodies following vaccination against SARS-CoV-2, primarily in subject seropositive for anti-N (i.e. with previous SARS-CoV-2 infection), whose antibody titre is similar to that of younger individuals. It is also evident that elderly adults, who have not contracted the virus naturally (anti-N negative), produce less anti-S antibodies than younger adults. This is the first report providing post-vaccination serological data in subjects aged 90 + years old to the best of our knowledge.

The features of this subpopulation must be considered in the strategy of organisation of the vaccination campaign against Sars-CoV-2. They are at high risk of developing severe COVID-19-related symptoms but leading an inactive and often isolated lifestyle it was assumed that they would have a fewer chance of contracting and transmitting the virus. For this reason, different strategies for administering vaccination to elderly adults have been adopted in other countries. For example, in the United States, the vaccine was given primarily to healthcare professionals and adults over the age of 65^17^. In China, however, until March 2021, the government did not recommend the vaccination of adults over 60, favouring preventive measures (i.e., social isolation, home confinement, and quarantine) to protect elderly adults^18^. In countries where priority has been given to preventive measures (i.e., social isolation, home confinement, and quarantine) to protect elderly adults^19^, the researchers interested in the ageing point out the harmful consequences of social isolation and loneliness on older peoples’ mental and physical health^20,21^.

In addition, very elderly adults hold different beliefs about COVID-19 vaccination. Among the main facilitators of vaccination, intentions are convenience (both individual and collective), psychological and physiological well-being, collective well-being, regulatory support referents and confidence in the government’s ability to get people vaccinated^22^. At the same time, the different barriers to vaccination intentions are vaccine ineffectiveness, side effects, safety, non-supporting regulatory referents, and the accessibility, accessibility, and availability of COVID-19 vaccines^22^.

The data obtained from the analysis of a cohort consisting of 97 subjects + 90 y/o suggest that SARS-CoV-2 viral-specific antibody response profiles are distinct in different age groups and that age-targeted strategies for vaccination management may be warranted. These data demonstrate that SARS-CoV-2 IgG antibody production differed in various age groups. Furthermore, other studies focused mainly on hospitalised elderly patients^23^; this study investigated the antibody responses to vaccination in non-hospitalised patients, thus indicating vaccination in relatively healthy subjects, such as elderly people who receive home care assistance.

This study obtained a comprehensive assessment of the SARS-CoV-2 antibody quantitative and qualitative profiles of very elderly adults, focusing on age and gender. Therefore, the data obtained show that the differences in the SARS-CoV-2 serology in the general population compared with elderly adults could be partly due to age-related immune responses.

This data analysis highlighted that sex is an essential factor to be considered when evaluating serostatus after SARS-CoV-2 infection and/or vaccination; even if previous infections and age represent the most critical determinants of anti-spike antibodies titre, sex significantly contributes to this value, especially among younger age categories^13^. This data is essential in managing factors that facilitate intentions to receive the vaccination (i.e., vaccine effectiveness), considering that the women respond differently to the COVID-19 pandemic regarding risk perception and behaviour than men^24,25^.

Another interesting data is that, even in elderly adults, higher levels of anti-SARS-CoV-2 IgG have been found in subjects who have contracted the virus and subsequently vaccinated, confirming what has already been found in the general population^26^.

A considerable amount of effort has been devoted to developing COVID-19 vaccines and promoting vaccination campaigns. As of 14 February 2022, 10.227.670.521 vaccine doses have been administered worldwide^1^. Therefore, it would be essential to optimise and standardise the administration strategy of vaccines to obtain the maximum possible efficacy. In many Western countries, the government focused on vaccinating elderly adults as soon as possible. For example, in the United States, adults over age 65 were the first group (along with healthcare providers) to receive the vaccines. In the United States, about 48 million (86.9%) of the population over 65 years of age have received at least one dose of the vaccine COVID-19, and about 42 million (76.5%) of the population is fully vaccinated by June 2021^27^. On the other hand, the Chinese government has recommended vaccination of adults over 60 only in March 2021^28^.

Consequently, vaccination coverage among elderly adults in China was drastically different from most other countries. China has not yet published official data on vaccination coverage among elderly adults. However, it is widely believed that the current vaccination coverage rate among elderly Chinese adults is critically low because elderly adults report a high level of doubt about the effectiveness of vaccines^22^. They have different perceptions of vaccination than the younger population; some elderly adults rely on traditional home health practices and healthy lifestyles as strategies for maintaining health^22^.

Moreover, several studies also indicate that risk perception and worry decrease with increasing age^29^. Older men show a lower perception of risk and are less inclined to implement changes in their health behaviour than women and young people^24^. All this most likely influenced older people’s perception of risk and behaviour and could also affect the turnout to vaccination. On the other hand, a limited number of studies have examined elderly adults’ vaccination intentions and the associated facilitators and barriers^22^, demonstrating that there are specific drivers (suffering from chronic diseases, having a medical professional talking through side effects and the importance of getting vaccinated) and barriers (concerns for vaccine effectiveness and side effects)^22^.

Finally, the SARS-CoV-2 antibody titre trend could support the strategies of administering an extra dose of vaccine as an additional (provided, as part of the primary vaccination cycle, for transplanted and immunocompromised subjects) or booster dose (provided, at least 6 months from the completion of the primary cycle, to the general population based on the epidemiological trend). In particular, the antibody titre could be used as a support index to establish the need for an extra dose, particularly in elderly people, whose humoral response to infections may be less efficient due to immunosenescence^30,31^ than those who had contracted the infection^26^.

## Conclusions

This study suggests that there are distinct SARS-CoV-2 viral-specific antibody response profiles that vary based on anti-N serostatus, age, and sex in the very elderly adults. Measurements of SARS-CoV-2 viral-specific antibody response profiles could also guide rational vaccine choice and deployment based on these variables. The present data claim that vaccination should be personalised (i.e., prioritisation strategies, administration of additional doses, facilitating intentions to receive the vaccination) and every therapy based on age-targeted strategies. Laboratory and clinical findings should support the definition of “fragile” people.

## Supplementary Information

Supplementary Information.

## Data Availability

The datasets generated and analysed during the current study are available in the OSFHOME repository, at the following link: https://osf.io/2a6pk/?view_only=728c766de3164b54900a418f9d26edb5.
