# Supplementary material for: Six months SARS-CoV-2 serology in a cohort of mRNA vaccinated subjects over 90 years old
Source: Sci Rep. 2022 Jul 20;12:12446. doi: 10.1038/s41598-022-15148-z (PMC9298708; doi:10.1038/s41598-022-15148-z)
Supplement: Supplementary file 1 — Supplementary Information. [file 41598_2022_15148_MOESM1_ESM.pdf]

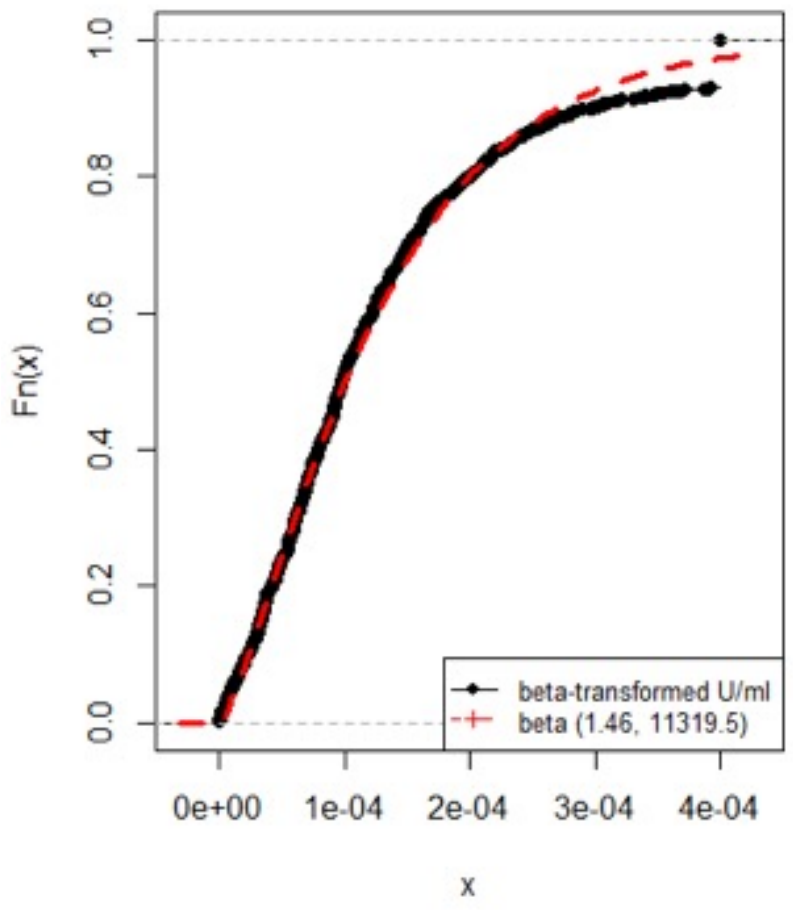

Supplementary Figure S1: **Anti-S antibody titer distribution at six months.**

Empirical cumulative distribution function of beta-transformed values of AntiS titer (U/ml) and theoretical beta distribution with alpha and beta parameters calculated on the observed values.
